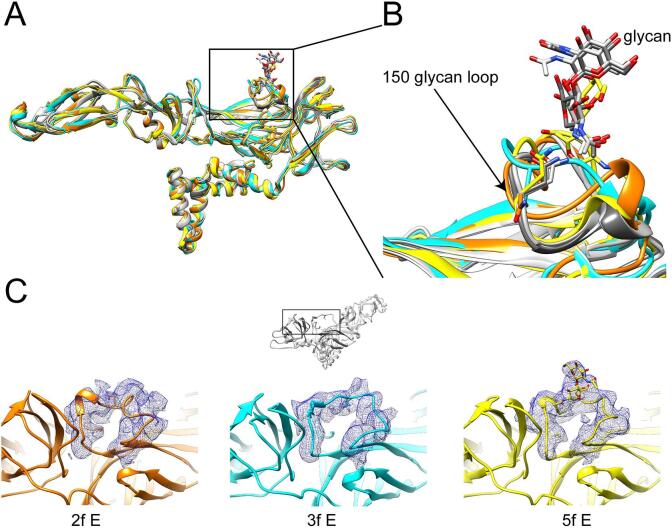# A complex between the Zika virion and the Fab of a broadly cross-reactive neutralizing monoclonal antibody revealed by cryo-EM and single particle analysis at 4.1 Å resolution

**DOI:** 10.1016/j.yjsbx.2020.100028

**Published:** 2020-06-17

**Authors:** Anu Tyagi, Tofayel Ahmed, Jian Shi, Shashi Bhushan

**Affiliations:** a School of Biological Sciences, Nanyang Technological University, Singapore; b Center for Bio-Imaging Sciences, National University of Singapore, Singapore; c Nanyang Institute of Structural Biology, Experimental Medicine Building, 59 Nanyang Drive, 636921, Singapore

**Keywords:** Zika virus, Cryo-electron microscopy, Fab, Neutralizing antibody

## Abstract

Zika virus (ZIKV) recently emerged as a major public health concern because it can cause fetal microcephaly and neurological disease such as the Guillain-Barré syndrome. A particularly potent class of broadly neutralizing antibodies (nAbs) targets a quaternary epitope located at the interface of two envelope proteins monomers, exposed at the surface of the mature virion. This “E-dimer-dependent epitope” (EDE), comprises the fusion loop of one monomer at the tip of domain II of E and a portion of the domains I and III of the adjacent monomer. Since this epitope largely overlaps with the binding site of the precursor membrane protein (prM) during Zika virion maturation, its molecular surface is evolutionary conserved in flaviviruses such as Dengue and Zika viruses, and can elicit antibodies that broadly neutralize various ZIKV strains. Here, we present a cryo-EM reconstruction at 4.1 Å resolution of the virion bound to the antigen binding fragment (Fab) of an antibody that targets this mutationally-constrained quaternary epitope. The Fab incompletely covers the surface of the virion as it does not bind next to its 5-fold icosahedral axes. The structure reveals details of the binding mode of this potent neutralizing class of antibodies and can inform the design of immunogens and vaccines targeting this conserved epitope.

## 1 Introduction

Zika virus (ZIKV) was first isolated in 1947 in Uganda from the serum of a sentinel rhesus monkey (Baud et al., 2017). Human infections with ZIKV were subsequently documented in 1952, both in Uganda and Tanzania (Baud et al., 2017, Galán-Huerta et al., 2016). Since then, sporadic ZIKV infections must have occurred, but the virus remained largely unnoticed. However, in 2007, a cluster of infections by ZIKV emerged in Yap Island in the Pacific Ocean, which was followed in 2013–2014 by a larger outbreak in French Polynesia, that included cases of the Guillain-Barré neurological syndrome (Baud et al., 2017). Moreover, in 2015, reports originating from Brazil linked ZIKV-infection with teratogenic effects in newborns such as microcephaly (Baud et al., 2017). Thus, in 2016, the magnitude of the outbreak and potential severity of the disease led the World Health Organization to declare ZIKV a “public health emergency of international concern” (Desai et al., 2017). The virus is primarily transmitted by *Aedes Aegypti* and *Aedes Albopictus* mosquitoes (Galán-Huerta et al., 2016), but other routes of human transmissions through body fluids such as semen have been identified. ZIKV belongs to the flavivirus genus within the *Flaviviridae* family (Galán-Huerta et al., 2016). Thus, ZIKV is closely related to other human pathogens of global concern such as dengue virus (DENV), Japanese encephalitis virus (JEV), West Nile virus (WNV), and Yellow fever virus (YFV) (Daep et al., 2014). The ZIKV genome is a positive-sense, single-stranded RNA molecule of about 11 kilobases that encodes three structural proteins: the capsid (C), membrane (prM), and envelope (E) glycoprotein, as well as seven non-structural proteins (NS1, NS2A, NS2B, NS3, NS4A, NS4B and NS5) (Wang et al., 2017). Phylogenetic analysis based on the NS5 gene sequence revealed three lineages of ZIKV: East African, West African and Asian (Faye et al., 2014, Musso, 2015, Robert et al., 2008). However, based on serum analysis, only a single ZIKV serotype has been identified so far (Dowd et al., 2016). The structure of the complete mature ZIKV was determined by cryo-EM, which revealed an overall architecture similar to mature DENV or WNV, however, the ZIKV virion is structurally more thermostable (Sirohi et al., 2016, Kostyuchenko et al., 2016). The outer icosahedral shell of the mature viral particle comprises 180 copies of the membrane (M) and the envelope (E) glycoprotein, which mediates attachment to the host cell and viral entry. The main antigenic determinant lies on the E protein, which is composed of three domains (labeled DI, DII and DIII) and is anchored in the viral lipid membrane via its C-terminal end (Sirohi et al., 2016). Accordingly, most virus-neutralizing antibodies target the E protein (Shi and Gao, 2017). A solvent exposed loop in the E protein from ZIKV with the sequence: 153VNDT156 bears a glycan N-linked to Asn154 (Sirohi et al., 2016). Via reverse genetics and structural studies, this “glycan loop” was proposed to play a major role in virulence and pathogenicity (Wang et al., 2017). Following large structural rearrangements that converts the E dimer lying flat on the virion surface into an upright trimer, the viral and endosomal membrane of the host cell merge (Lee et al., 2018). In the trimeric fusogenic conformation of E, the fusion loop (FL) located at the extremity of DII inserts in the target membrane (Lee et al., 2018). At neutral pH, the FL is buried at the interface with another E monomer in a head-to-tail dimer (Lee et al., 2018). In the absence of an FDA-approved vaccine or antiviral drugs against ZIKV, a significant effort has been invested by the scientific community to identify potent nAbs for immunotherapy (Sun et al., 2017). Several potent nAbs target the DIII of the E-protein with epitopes mapping to the lateral ridge of E, its C-C’ loop and β-strand A, which is exposed at the surface of the virion (Screaton et al. 2015, Nybakken et al., 2005, Stettler et al., 2016, Robinson et al., 2015, Robbiani et al., 2017, Zhao et al., 2016, Sukupolvi-Petty, 2010, Tharakaraman et al., 2013). Many nAbs, with various levels of neutralizing activity, bind to DI and DII of the E-protein, where they target the DI-DII hinge, the FL or the BC loop of DII (Dai et al., 2016, Throsby et al., 2006, Oliphant et al., 2007, Wang et al., 2016, Smith et al., 2013, Fibriansah et al., 2014, Deng et al., 2011, de Alwis et al., 2012, Long et al., 2019).

Particularly interesting is a group of antibodies that bind to quaternary epitopes comprising residues from two adjacent E-protein monomers (named “E-dimer-dependent epitope” or EDE): while some of these antibodies are serotype specific (Teoh et al., 2012, Fibriansah et al., 2015a, Fibriansah et al., 2015a), others can broadly neutralize both ZIKV and DENV (Dejnirattisai et al., 2015, Fernandez et al., 2017). Their epitopes, which comprises the FL of DII, is highly conserved across the four DENV serotypes and also in various ZIKV isolates, presumably because several residues in the epitope also serve as an anchoring site for prM, during virus maturation through the Golgi network (Rouvinski et al., 2015, Barba-Spaeth et al., 2016). This quaternary epitope is recognized by monoclonal antibodies (mAbs) C8 and ZAb_FLEP (for “Fusion loop epitope-proximal”) (Barba-Spaeth et al., 2016, Tharakaraman, et al., 2018). Recent studies have reported the high neutralization potency of ZAb_FLEP and C8 (Barba-Spaeth et al., 2016, Tharakaraman, et al., 2018). A preliminary mapping of the epitope bound by ZAb_FLEP was performed using cryo-EM, leading to a structure of its Fab bound to ZIKV at 9.7 Å resolution (Tharakaraman et al., 2018). However, the limited resolution attained in this study precluded a complete characterization of the epitope. Here we present a three-dimensional cryo-EM reconstruction of ZIKV bound to 120 copies of the Fab from ZAb_FLEP at an average resolution of 4.1 Å. At this resolution, the cryo-EM density map enabled us to visualize details of how the quaternary epitope is recognized in the context of the complete mature flavivirus virion. Our studies provide structural insights at near atomic resolution into ZIKV neutralization by the mAb ZAb_FLEP, which will hopefully guide the design of improved immunogens to accelerate the development of vaccines against ZIKV and other flaviviruses.

## 2 Results

### 2.1 Structure determination

**Fig. 1:**
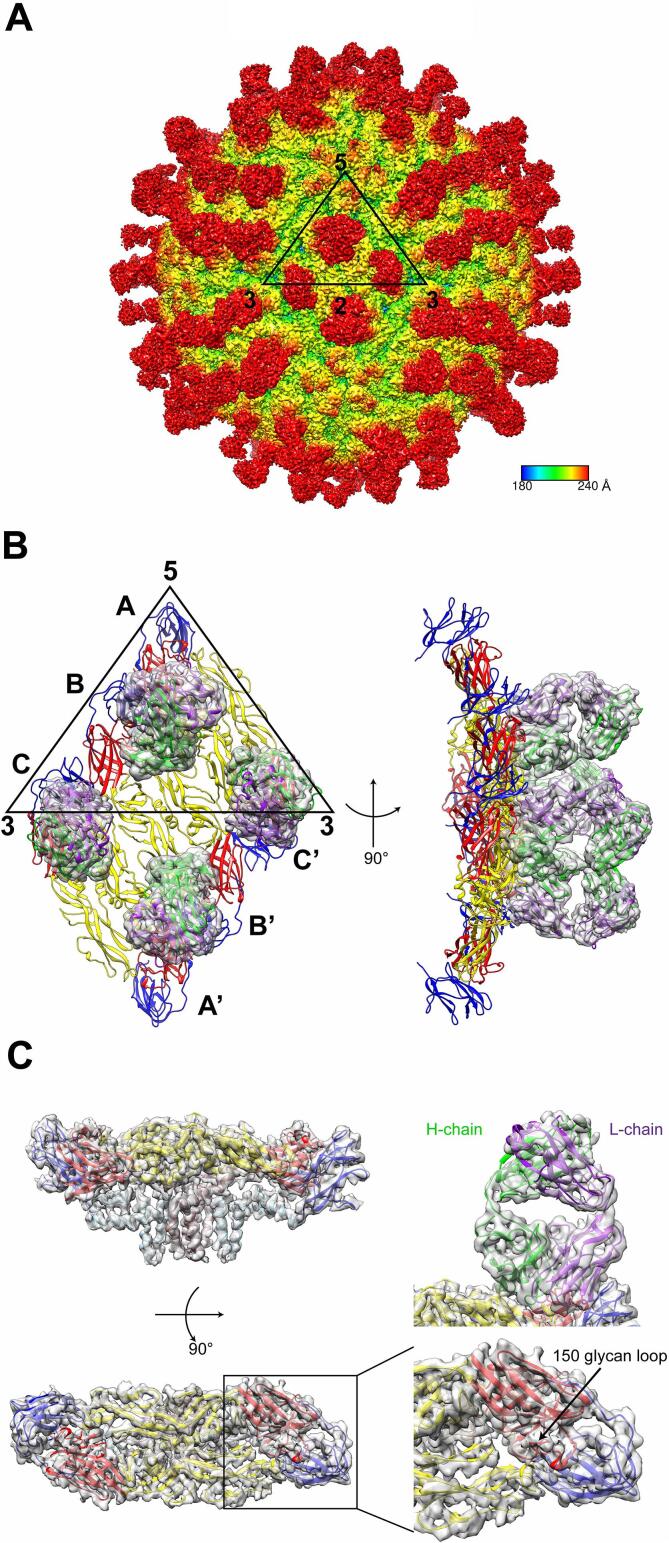
The Cryo-EM structure of ZIKV complexed with ZAb_FLEP. (A) Cryo-EM map of ZIKV bound with ZAb_FLEP. The ZIKV-Fab surface is shown in radial colouring. The icosahedral 2, 3 and 5 fold axes are marked and a representative asymmetric unit is shown as a black triangle. (B) Top view (left panel) and side view (right panel) of two Fab ZAb_FLEP molecules bound per asymmetric unit. The three individual E proteins in an asymmetric unit are labeled as A, B, and C. The same molecules from an adjacent asymmetric unit in a raft structure are labeled as A′, B′, and C′. DI, DII, and DIII of E protein in ribbon representation are colored in red, yellow, and blue, respectively. The heavy chain and light chain of Fab ZAb_FLEP are represented in green and purple ribbon covered with transparent density. (C) Fitting of the E protein and antibody model into the density map. The sideview and topview of E proteins are shown in the upper left and lower left panel, respectively. 150 glycan loop which is unique for ZIKV is enlarged in lower right panel. The heavy chain (green) and light chain (purple) of antibody are shown in the upper right panel. Color scheme of E proteins DI, DII and DIII domain is the same as in (B). The stem and transmembrane region of the E protein is coloured in light blue, whereas the same region in the M protein is coloured in rosy brown. (For interpretation of the references to color in this figure legend, the reader is referred to the web version of this article.)

**Supplementary figure 1:**
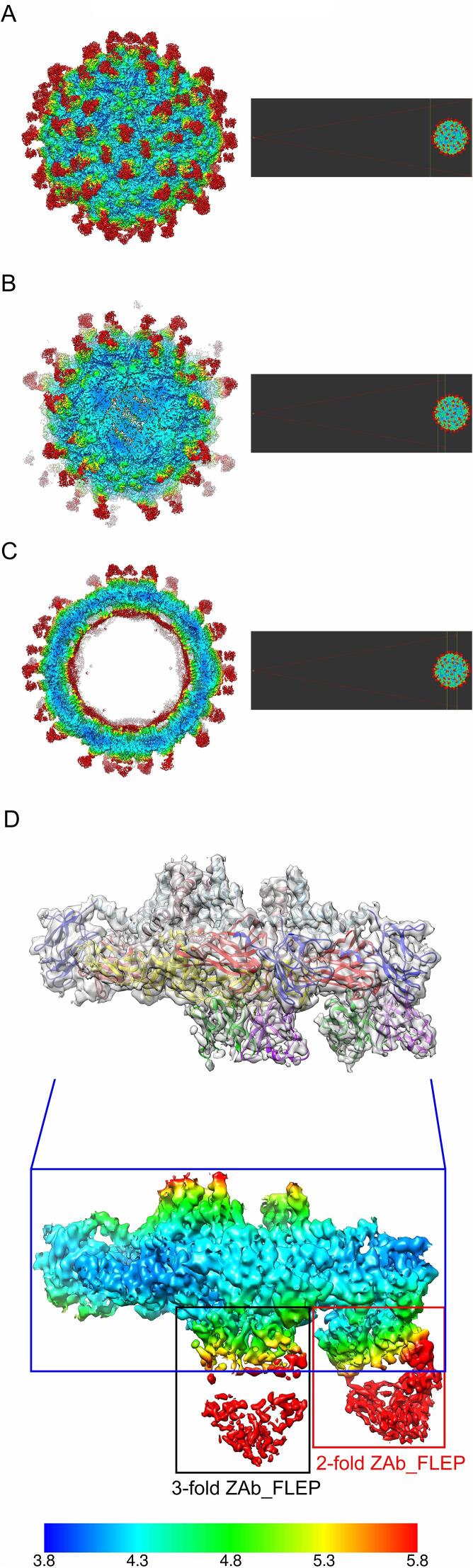


We used cryo-EM and single particle analysis for structure determination. The complex was prepared by incubating purified ZIKV with the Fab from mAb ZAb_FLEP obtained following papain digestion, as described before (Zhang et al., 2016). Vitrified complex was imaged on a FEI Krios electron microscope operating at 300 kV using a Falcon II direct electron detector in movie mode. A representative cryo-EM micrograph is shown in Fig. S1A. Data were processed with Relion 2 image processing package (Scheres, 2012), which resulted in an electron density map with an average resolution of 4.1 Å, based on the 0.143 Fourier Shell Correlation criteria (Fig. S1B). However, the local resolution varies throughout the volume of the reconstruction (Fig. S2). The virion is composed of an icosahedral shell consisting of 180 copies each of the E protein and M protein, which are both anchored in the viral envelope via their C-terminal *trans*-membrane regions (Fig. 1A and C). The E-proteins that bind the receptor and catalyze membrane fusion, are exposed at the surface of the viral particle and the M proteins are buried beneath the layer of E proteins (Fig. 1C). The M protein comprises a N-terminal loop, a stem region and *trans*-membrane region that anchor the M protein in the lipid membrane, that are all clearly visible in the cryo-EM density map (Fig. 1C). Compared to the study published previously at 9.7 Å resolution, the present structure reveals more details in terms of moderately resolved side chains in some of the α-helices in both proteins E and M, and discernible individual β-strands in most regions concerning protein E (Fig. S3). The surface of the virion can be described in terms of 60 repeats of an asymmetric unit containing three E-proteins. Three such head-to-tail E dimers constitute “the raft” (Fig. 1B) and 30 rafts are present at the virion surface. The ectodomain of the E-protein is comprised of three regions labeled DI, DII and DIII that are rich in β-sheet, together with a helical stem and *trans*-membrane region (Fig. 1C).

A distinguishing feature of ZIKV is an insertion within the glycosylated loop of E named the “glycan loop” located in DI. This loop protrudes from the mature virion surface (Fig. 1C and S3). The motif (residues 146-SQHSGMIVNDTGHETDE-162) is in close proximity to the E protein dimer interface and the fusion loop of the adjacent E protein. The glycan loop comprises five more residues in the E protein from ZIKV, compared to the corresponding loop in DENV1-4 (Fig. S5). Interestingly, this loop is also longer in other neurotropic viruses such as WNV or JEV (Long et al., 2019). Thus, glycosylation at the N154 site in ZIKV E protein was proposed to play an important role in virus entry and pathogenicity (Sirohi et al., 2016, Kostyuchenko et al., 2016).

A total of 120 Fab molecules are attached to the virion with good occupancy, near the 2-fold and 3-fold axes (Fig. 1A and B). However, the Fab does not bind at sites near the twelve 5-fold axes (Figs. 1A and S6). While the complementarity-determining regions (CDRs) of the Fab that are brought in contact with the epitope on the viral particle are well resolved, their constant dimers appear more flexible (Fig. S2D). Despite the relatively moderate resolution of the present reconstruction, the abundance of aromatic amino acids in the CDR regions of the Fab allowed side-chain building at the virus-antibody interface with confidence. Overall, the electron density of the Fab is better resolved at the 2-fold axes compared to the Fab located next to the icosahedral 3-fold axes (Fig. S2D). Therefore, building of the model for the Fab was performed first at 2-fold axes and this model was subsequently placed at the 3-fold axes, where manual adjustments were performed (Fig. S2D).

### 2.2 Epitope recognition

**Fig. 2:**
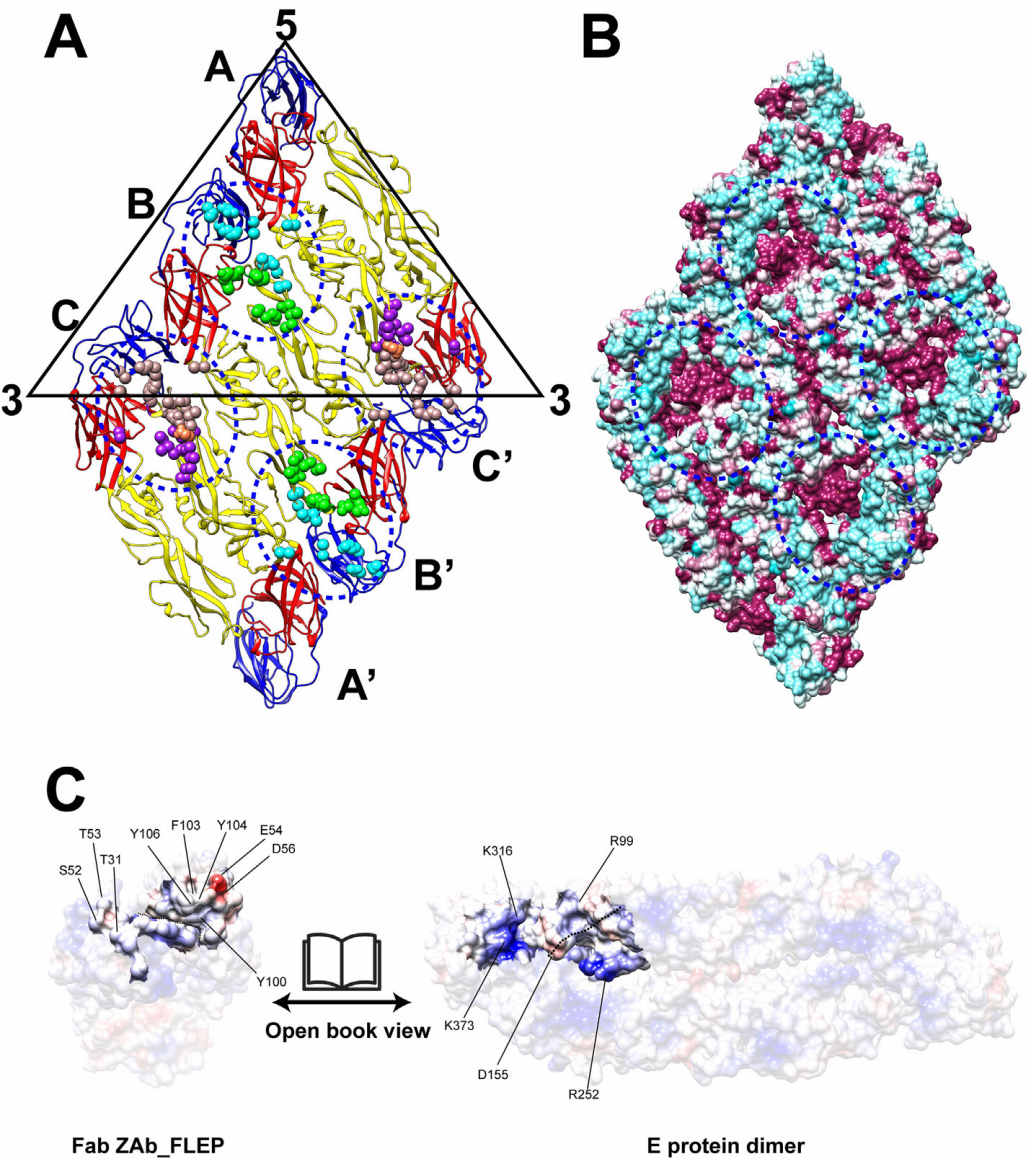
The ZAb_FLEP epitopes on the ZIKV-ZAb_FLEP complex structure. (A) The ZAb_FLEP epitope residues (circled by blue dots) in an E protein raft identified by 2P2I inspector v2.0 server (http://2p2idb.cnrs-mrs.fr/2p2i_inspector.html). The epitope residues for heavy chain and light chain of 2-fold antibody binding are shown as green and cyan spheres. The common epitope residues between heavy chain and light chain of 2-fold antibody are shown in sienna spheres. The epitope residues for heavy chain and light chain of 3-fold antibody binding are shown as purple and rosy brown spheres. The common epitope residues between heavy chain and light chain of 3-fold antibody are shown in coral spheres. (B) The conservation of epitope in an E protein raft. The ZIKV ZAb_FLEP epitope residues that are conserved and non-conserved when compared to DENV1-4, ZIKV (MR766) and ZIKV(PF13) are coloured from maroon to cyan. (C) Charge complementarity of the ZAb_FLEP intra-dimer B-B’ epitope with the Fab paratope. Positive, negative and neutral charges are coloured in blue, red and white, respectively. Possible interacting residues are labelled. (For interpretation of the references to color in this figure legend, the reader is referred to the web version of this article.)

The Fabs are clustered next to the 2- and 3-fold icosahedral axes, where they each bind to residues projecting from two adjacent E monomers with a marginal contribution of a third E monomer (Fig. 2). One Fab binds near the 2-fold axis to two E monomers forming a head-to-tail dimer labeled B and B’, with just an additional residue Asn52 from the third adjacent E protein labeled “A” participating in the interaction (Fig. 2A). The regions of the E protein that are brought in contact with the Fab are DIII of monomer B, DII including the fusion loop of monomer B’, as well as the hinge region between DI and DII of monomer A. Another Fab is bound next to the 3-fold icosahedral axis, making several contacts with monomers A’ and C (that form the head-to-tail dimer), with also Asn52 from monomer B establishing a single contact (Fig. 2A). Therefore, most of the epitope is contributed by residues from the E-protein intra-dimers named B-B’ (near the 2-fold axis) and A′–C or A–C′ (near the 3-fold axis) (Fig. 2A). Remarkably, as discussed below, this arrangement of Fab leaves unoccupied the corresponding quaternary epitopes near the 5-fold vertices (Fig. S6). For each of the Fab binding near the 2-fold or 3-fold axes, we give pictorial representations to enlist all the residues that interact with the virion (Figs. S7 and S8). Overall, the binding mode of the Fab at the 2- and 3-fold axes is similar but not identical as shown in Figs. 1, S7 and S8.

The paratope for the Fab binding near the 2-fold axis comprises five of its six CDRs: for the light chain, L1 (residues 28 and 31–32), L2 (residue 52 and 53), L3 (residues 92–95) (Fig. S7C–E) are involved in direct contacts with the epitope, whilst for the heavy chain, H2 (residues 54 and 56–57, 59–60, 65) and H3 (residues 100 and 102–104, 106, 108) (Fig. S7A and B) participate in interactions. An additional interaction is observed with residue Ser63 from the framework region 3(FR3) (Fig. S7E). The paratope for the Fab located near the 3-fold axis is slightly different than 2-fold Fab and is comprised of: L1 (residues 28 and 31–32), L2 (residues 50, 52–53), L3 (residues 92–94) (Fig. S8C–E), H2 (residues 54, 56–58, 60) and H3 (residues 100, 103–104, 106) (Fig. S8A and B). Moreover, an additional interaction is also seen with residue Arg66 from the framework region 3 (FR3) (Fig. S8E). Participation of residues from framework regions has been documented for other antigen–antibody complexes (Kostyuchenko et al., 2016). Interactions between the Fab and E protein on ZIKV in one asymmetric unit consists of 11 hydrogen bonds and 3 salt bridges, as analyzed by default parameters set in LigPlot + v2.2 (Laskowski and Swindells, 2011) (Figs. S7 and S8). As seen in Fig. 2C, both shape and charge complementarity play an important role in complex formation: for example, in 2-fold Fab, the negatively charged E54 of the heavy chain interacts with R252 of the E protein (Figs. 2C and S7A), while hydrogen bonds and hydrophobic interactions are formed by T31, S52 and T53 of the light chain with K316 and K373 of the E protein (Figs. 2C and S8E).

### 2.3 The Fab does not bind around the 5-fold axes

**Fig. 3:**
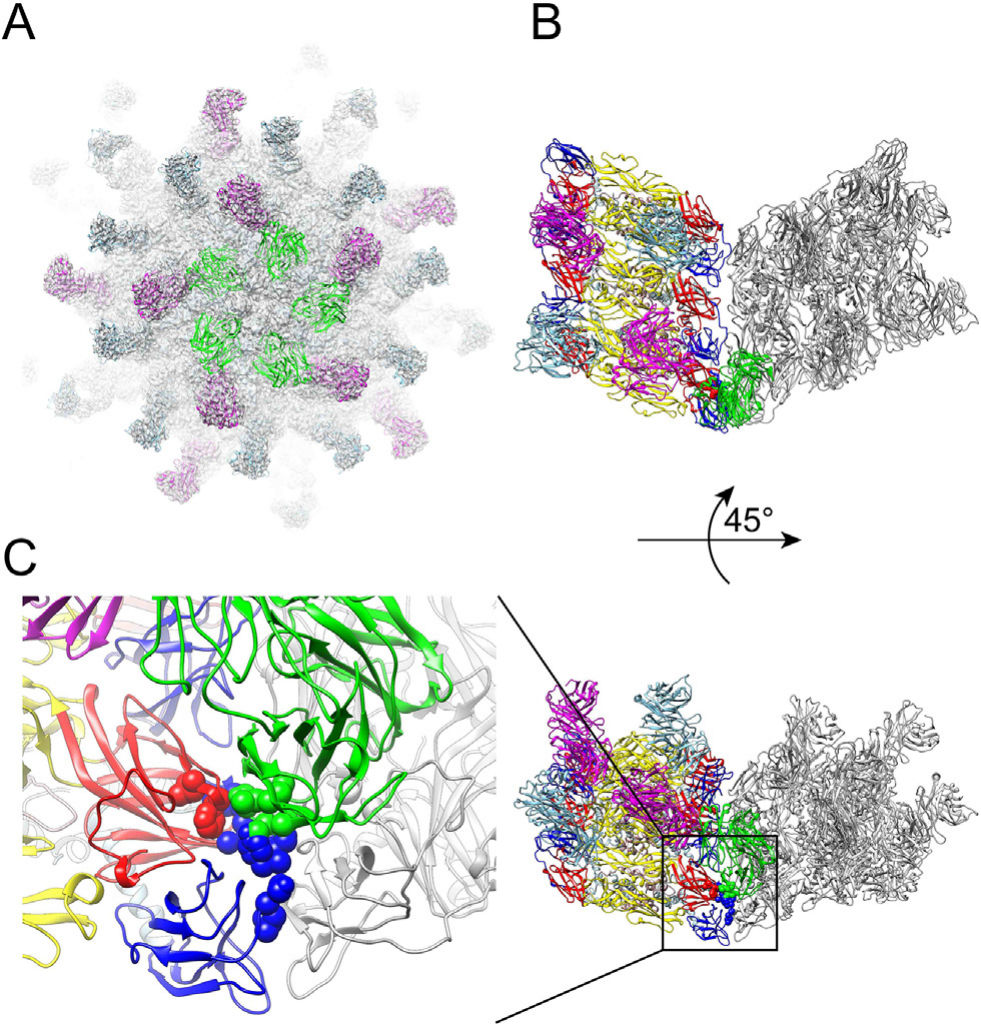
Binding of ZAb_FLEP Fabs at the 5-fold site are hindered by steric clashes with E protein in nearby raft. (A) Fab docking on 5-fold axis. The figure shows a surface representation of the ZIKV/ZAb_FLEP cryo-EM map oriented down an icosahedral 5-fold axis. The Fabs bound at the 2-fold, 3-fold and 5-fold sites are colored in magenta, skyblue and green. Binding of Fabs to the 5-fold sites related by icosahedral 5-fold symmetry does not lead to steric hindrance between symmetry related Fabs. (B) Topview and (C) 45° Sideview of steric clashes between 5-fold Fab and E protein in nearby raft. Left panel in (C) shows enlarged sideview of steric clashes between 5-fold Fab and E protein in nearby raft. For clarify, only one raft in gray bound with 5-fold Fab in green and its nearby raft colored same with Fig. 2A are shown. Possible residues involved in steric clash are shown in space-filling representation. (For interpretation of the references to color in this figure legend, the reader is referred to the web version of this article.)

No Fab binding was observed at epitopes around the 5-fold axes of the viral particle (Figs. 1A and S6). Hence the virion is covered by only 120 Fab molecules, instead of the 180 theoretically- possible Fab molecules, for full occupancy. Two mutually non-exclusive explanations are possible: (i) Clustering of Fabs around the 5-fold axes could lead to steric hindrance between neighboring bound Fab molecules, either with symmetries generated by the 5-fold axis or Fabs located near the 2- and 3-fold axis. (ii) The local conformation of the epitope is presented near the 5-fold vertices differently, compared to its configuration at the 2- and 3-fold axes, precluding binding with high occupancy. To address this question, we artificially built a virion covered with full occupancy having 180 bound Fab molecules. To create this artificial complex, a Fab molecule located at 2-fold axis bound to dimer B-B’ was docked at the 5-fold axis using a dimer superposition onto AC’ (Fig. 3). As a result, no steric hindrance is observed between Fab molecules bound around the 5-fold vertices, nor with Fab molecules near the 2-fold axis (Fig. 3A). Instead, the local environment of Fabs binding near the 5-fold vertices clearly differs from 2-fold or 3-fold binding sites, as it encompasses inter-raft E proteins and not only intra-raft E proteins, to which Fab molecules bind near the 2-fold or 3-fold axes (Fig. 3B). Fab molecules docked near the 5-fold closely approach inter-raft E proteins, which results in steric hindrances with residues from the epitope, thus preventing Fab (and also presumably a whole antibody) binding at these locations (Fig. 3C). In conclusion, variation in the local conformation of the epitope that is presented near the 5-fold vertices explains lack of binding at these positions.

### 2.4 Mechanism of neutralization

The epitope is distributed on the three domains of E protein and includes residues crucial for ZIKV attachment and entry via low-pH induced fusion. DIII of DENV E protein has been proposed to participate in receptor binding, but its role in ZIKV attachment is elusive (Watterson et al., 2012, Kaufmann and Rossmann, 2011). The hinge between DI and DII allows movements important during virus entry (Modis et al., 2004). Most importantly, the epitope comprises the FL at the extremity of DII, which is essential for entry, as it inserts into the endosomal membrane triggering fusion (Modis et al., 2004). ZIKV has a single glycosylation site at Asn154 in DI, which projects from the surface. The “150 loop” (residues 146–162) is longer compared to the E protein from other flaviviruses such as DENV (Fig. S5A). Dendritic cell-specific intercellular adhesion molecule 3-grabbing non-integrin (DC-SIGN) and the mannose receptor are putative viral attachment proteins for DENV, that bind to DENV glycans (Navarro-Sanchez et al., 2003, Tassaneetrithep et al., 2003, Richter et al., 2014, Pokidysheva et al., 2006, Miller et al., 2008). Thus, the Asn154 glycosylation may function as an attachment site of ZIKV to host cells. The glycan loop region is variable amongst ZIKV isolates and also other flaviviruses, which indicates that differences in this region may influence virus transmission and disease severity (Shi and Gao, 2017). When Fab ZAb_FLEP binds to the glycan loop, including the Asn154 glycosylation site, some conformational changes are visible as depicted in Fig. S3B. A superposition of unbound E protein monomers ((PDB ID: 6CO8) at the 2-fold, 3-fold and 5-fold axes E protein shows good structural conservation of the150 glycan loop (Fig. S3A). However, overlay with the corresponding 150 loop at the 2-fold and 3-fold axes in the present bound structure reveals conformational changes upon Fab binding (Fig. S3A). This is consistent with the observation that the glycan loop at the 5-fold vertices (where no Fab bind) conform well to the consensus unbound 150 loop, thus ruling out over-interpretation due to the limited resolution of the present reconstruction. Of note, clear electron density was observed for the N-linked glycan at the 5-fold E protein, whereas no clear density is visible for glycan in the E protein located near the 2- and 3-fold, which could therefore not be modeled (Fig. S3C). We speculate that Fab ZAb_FLEP, which targets this 150 glycan loop, may prevent ZIKV attachment to host cells by partially masking the glycan loop.

## 3 Discussion

Recent studies have identified several nAbs against ZIKV infection (Shi and Gao, 2017). Studies of nAbs bound to the virion using either cryo-EM or X-ray crystallography, have revealed the molecular basis of antibody-mediated neutralization against ZIKV (Shi and Gao, 2017). Antibodies that neutralize flaviviruses are usually divided into three major class: (*i*) one class targets the fusion loop epitope (FLE) centered on the hydrophobic fusion peptide on E protein, which is used by the virus to fuse viral and host cell membranes, in the acidic endosomal compartment; (*ii*) the second class targets an E dimer-dependent epitope (EDE), which is also the site of interaction of the envelope protein dimer with the precursor membrane (prM) protein, during virus maturation; (*iii*) a third class called ZIKV-specific neutralizing antibody (Shi and Gao, 2017).

Rey and collaborators have proposed two subgroups for EDE antibodies depending on the state of glycosylation of the 150 loop: the EDE1 group binds regardless of the glycosylation state of Asn153 in DENV while the EDE2 group requires glycosylation at position N153 in DENV for efficient binding (Dejnirattisai et al., 2015, Rouvinski et al., 2015, Barba-Spaeth et al., 2016).

The first reported nAb against ZIKV was 2A10G6, which targets the conserved FLE and protects broadly against flaviviruses (Dai et al., 2016, Deng et al., 2011). nAb 2A10G6 has broad cross-reactivity with DENV1-4, YFV, WNV, JEV, and TBEV. The epitope of 2A10G6 was mapped to the highly conserved flavivirus fusion loop peptide ^98^DRXW^101^ motif, a region essential for membrane fusion between the virus and the cell (Deng et al., 2011).

Subsequently, a subset of neutralizing antibodies (such as mAb C8 from the EDE1 class and A11 from the EDE2 class) were isolated from patients with dengue; Remarkably these mAbs also potently neutralize Zika virus (Barba-Spaeth et al., 2016). Both C8 and A11 are potent neutralizers of the ZIKV/DENV super serogroup (Barba-Spaeth et al., 2016). They bind to the E-dimer quaternary structure-dependent epitopes in the conserved region of ZIKV and DENV, which is also the interaction site of E-dimer with the precursor membrane protein (prM) during virus maturation (Barba-Spaeth et al., 2016).

In contrast to these flaviviruses cross-reactive nAbs, three ZIKV-specific antibodies (Z20, Z23 and Z3L1) isolated from a patient with Zika, show potent neutralizing activity without cross-reactivity to DENV1-4 (Wang et al., 2016). Structural studies indicated that these antibodies bound to different viral epitopes, suggesting that they could be used in the form of a therapeutic cocktail (Wang et al., 2016). By immunizing mice, four ZIKV-specific (ZV-48, ZV-54, ZV-64 and ZV-67), targeting distinct spatially epitopes in DIII of the envelope protein, have been isolated (Low et al., 2006). Combining with *in vivo* passive transfer studies, these results indicate that E-DIII-targeting antibodies could be ZIKV-specific nAbs (Low et al., 2006). Another study showed that an E-DIII-specific antibody could protect mice from a lethal ZIKV infection, further illustrating the potential for antibody-based therapy (Stettler et al., 2016).

In addition to neutralizing ZIKV, ZAb_FLEP cross-reacts and neutralizes the four serotypes of DENV (Tharakaraman, et al., 2018, Dejnirattisai et al., 2016). Sequence and structure comparison of our structure with DENV and other ZIKV strain shows the structural basis of cross-neutralization by ZAb_FLEP (Figs. 2B and S5A). Moreover, residues conserved between ZIKV and DENV reside in the epitope of ZAb_FLEP as shown in Fig. 2B. ZAb_FLEP binds to the quaternary-dependent epitopes in the conserved region of DENV and ZIKV, including: (*i*) the bc loop, the fusion loop, d strand, and the ij loop in DII of one E protein; (*ii*) the DI-DII hinge of another inter-dimer E protein. These conserved epitopes are also the site of interaction between the E protein dimer and the prM protein during virus maturation. Thus, conservation of this quaternary-dependent epitopes in terms of both its sequence and structure accounts for the cross-reactivity of ZAb_FLEP for ZIKV and DENV.

**Fig. 4:**
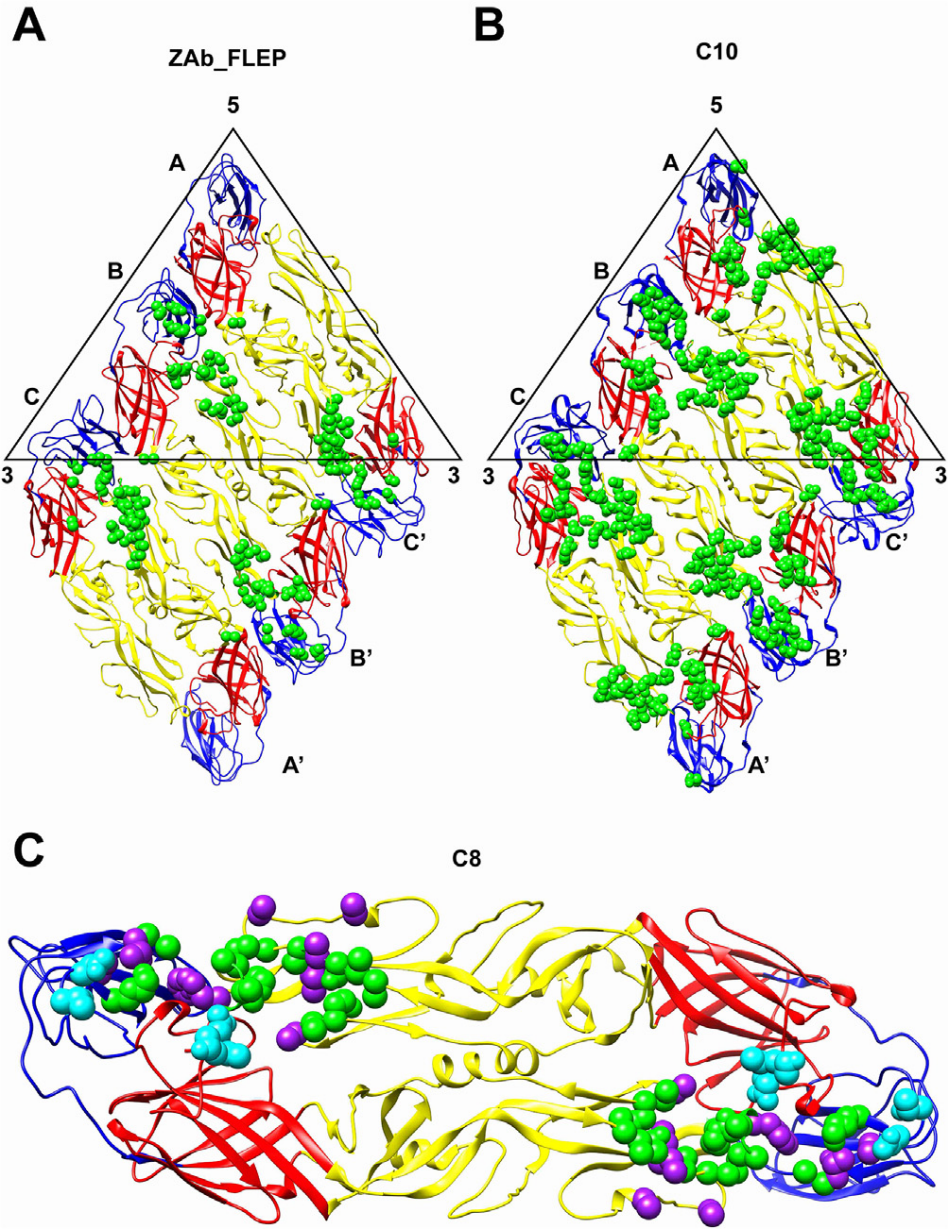
Comparison of quaternary structure-dependent epitopes on E protein. (A-B) Comparison of epitopes on E protein raft bound by ZAb_FLEP (A) and C10 (PDB ID: 5H37) (B). (C) Comparison of epitopes on intra-dimer E protein bound by ZAb_FLEP and C8 (PDB ID: 5LBS). The intra-dimer B-B’ epitope by ZAb_FLEP and the epitope in the crystal structure of soluble E protein bound with C8 are shown. The common epitope residues for both, the unique epitope residues for ZAb_FLEP and C8 are shown in green, cyan and purple spheres. (For interpretation of the references to color in this figure legend, the reader is referred to the web version of this article.)

We also compared the binding modes of Fab C8, Fab ZAb_FLEP and Fab C10 that belongs to EDE1 epitope; as cryo-EM structure of ZIKV bound with C10 is available, a comparison of binding mode on virion between ZAb_FLEP and C10 was also performed (Fig. 4B). Fab C10 binds at 2-fold, 3-fold and 5-fold axes whereas Fab ZAb_FLEP only binds at 2-fold and 3-fold axis. And the 5-fold epitope of C10 engages inter-raft E protein, which is absent in epitope of ZAb_FLEP (Zhang et al., 2016). Furthermore, the epitopes for 2-fold and 3-fold C10 are also much larger than ZAb_FLEP (Fig. 4B).

A footprint comparison between Fab ZAb_FLEP and the previously published crystal structure of ZIKV recombinant E protein dimer complexed with Fab C8 (Fig. 4C) shows a large overlap of their epitopes. However, the epitope of Fab C8 is larger than the intra-dimer epitope of ZAb_FLEP. The unique epitope residues of Fab C8 are Q77, D83, W101, K251 from DII of one E protein and Q331 from DIII of adjacent E protein, whereas the only unique epitope residue of ZAb_FLEP is N371 from DIII of one E protein. Furthermore, our cryo-EM structure also shows the interactions of ZAb_FLEP with other quaternary structure-dependent epitope at the inter-dimer interfaces, which were not observed in the crystal structure. As a result of this extended interaction network, the E proteins at the virus surface are locked together and presumably lose the dynamics necessary for infectivity, which is crucial for the neutralization mechanism.

An interesting question relates to how a complete IgG molecule would bind to the virion? Given the separation between Fab molecules observed in the present structure, it is likely that one Fab binding near the 3-fold is associated with another Fab near the 2-fold axis. This however leaves two pairing possibilities as illustrated in Fig. 2. Not only would bivalent binding significantly cover the raft with a layer of IgG molecules, presumably preventing receptor attachment and conformational changes needed for viral fusion, but it could also induce significant structural strain on the viral particle.

Which conclusion can be inferred from the comparison of these various nAbs in terms of vaccine development? Taken together, EDE1 antibody appear to be better suited to inspire the development of an epitope-focused vaccine for viruses in the ZIKV/DENV super serogroup than FLE antibodies, which induces poorly neutralizing and strong antibody-dependent enhancement (ADE) infection (Dejnirattisai et al., 2015, Goncalvez et al., 2007, Li et al., 2013). Moreover, EDE1 antibodies are better than EDE2 antibody which require the glycosylated 150 loop, where glycosylation is not always present as demonstrated by their poor affinity and strong ADE induction (Dejnirattisai et al., 2015, Barba-Spaeth et al., 2016).

In summary, our cryo-EM structure of ZIKV bound with ZAb_FLEP reveals a unique binding mode of potent EDE1 neutralizing antibody against ZIKV. These structural studies provide a molecular basis to develop immunogens able to elicit broadly neutralizing mAbs that could be used for vaccination against both DENV and ZIKV.

## 4 Methods

### 4.1 Sample preparation and cryo-electron microscopy

Purified Fab in buffer (15 mM Tris-HCl pH 8, 150 mM NaCl) and ZIKV virions in buffer (10 mM Tris-HCl pH 8.0, 120 mM NaCl and 1 mM EDTA) were mixed with a E protein: Fab (ZAb_FLEP) molar ratio of 1:2 as described earlier (Zhang et al., 2016). 2.5 µl of virus-Fab complex was applied onto glow-discharged 2-nm carbon coated holey grids (R2/2, Quantifoil GmbH, Germany) and incubated for 30 sec. Grids were manually blotted for 2 sec at RT and flash frozen in liquid N2-cooled liquid ethane as described earlier (Tharakaraman et al., 2018). Grids were loaded to a FEI Krios cryo-transmission electron microscope, operated at 300 kV and equipped with a back-thinned Falcon II direct electron detection device. A total of 7387 micrographs were recorded in a movie mode as a sets of 19 frames (total dose of 48 electrons per Å^2^) at a calibrated magnification of 109,375 resulting in a pixel size of 1.045 Å on the object scale and at defocus values from 0.7 to 3.5 μm.

### 4.2 Image processing

7222 micrographs were selected for data processing after removing micrographs with significant drift and astigmatism. Whole-image drift correction was carried out using motioncorr (Mindell and Grigorieff, 2003). CTF estimation was performed with CTFFIND3 (Tang et al., 2007). Particles were picked from the micrographs using EMAN2 (Emsley et al., 2010). Data was processed with RELION 2. In total, 64,789 particles were subjected to reference-free 2D classification to discard bad particles. 42,252 particles selected after 2D classifications were used for 3D classifications using a 60 Å filtered Zika EM map (EMD-8116) (Sirohi et al., 2016). A subset of 4610 particles was identified after 5 rounds of 3D classifications which yielded an EM density map at 4.1 Å resolution with icosahedral symmetry according to the gold-standard FSC = 0.143 criterion.

### 4.3 Structure fitting, refinement and analysis

Fab C8 (PDB ID: 5LBS) fitted quite well in our density and was used for initial model building. The variable domain of bound Fabs closest to the icosahedral 3-fold and 2-fold vertex were manually modelled in COOT (Adams et al., 2010) and refined with the real space refinement feature in PHENIX (Pettersen et al., 2004) iteratively. The coordinates of the E proteins obtained from a 3.1 Å resolution cryo-EM map of ZIKV (PDB ID: 6CO8) were fitted into the map and refined in real space using PHENIX. The figures were prepared with the Chimera (Schrodinger, 2015) and PyMOL (Basse, et al., 2013). The molecular contacts were analyzed using the 2P2I inspector v2.0 server (Chojnacki et al., 2017) and LigPlot + v2.2 (Laskowski and Swindells, 2011). The superpositions of structures were performed using the program Chimera (Schrodinger, 2015). Protein sequence alignments were conducted using the web server ClustalO (Robert and Gouet, 2014). The alignment figures were prepared using the web server ESPript (Kucukelbir et al., 2014). EM map and atomic model have been deposited with the Electron Microscopy Data Bank and Protein Data Bank under accession codes of EMD-30337 and 7CBP.

## CRediT authorship contribution statement

**Anu Tyagi:** Writing - review & editing. **Tofayel Ahmed:** Writing - review & editing. **Jian Shi:** Methodology. **Shashi Bhushan:** Conceptualization, Writing - review & editing.

## Declaration of Competing Interest

The authors declare that they have no known competing financial interests or personal relationships that could have appeared to influence the work reported in this paper.

## Figures and Tables

**Supplementary figure 2:**