# Supplementary figures and images for: A complex between the Zika virion and the Fab of a broadly cross-reactive neutralizing monoclonal antibody revealed by cryo-EM and single particle analysis at 4.1 Å resolution
Source: J Struct Biol X. 2020 Jun 17;4:100028. doi: 10.1016/j.yjsbx.2020.100028 (PMC7337043; doi:10.1016/j.yjsbx.2020.100028)

# Fig S1

A

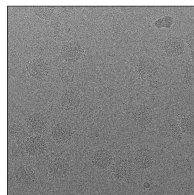

B

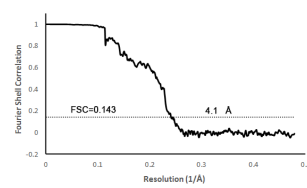

Supplement: Supplementary data 1 [file mmc1.pdf]

Figure S4

A

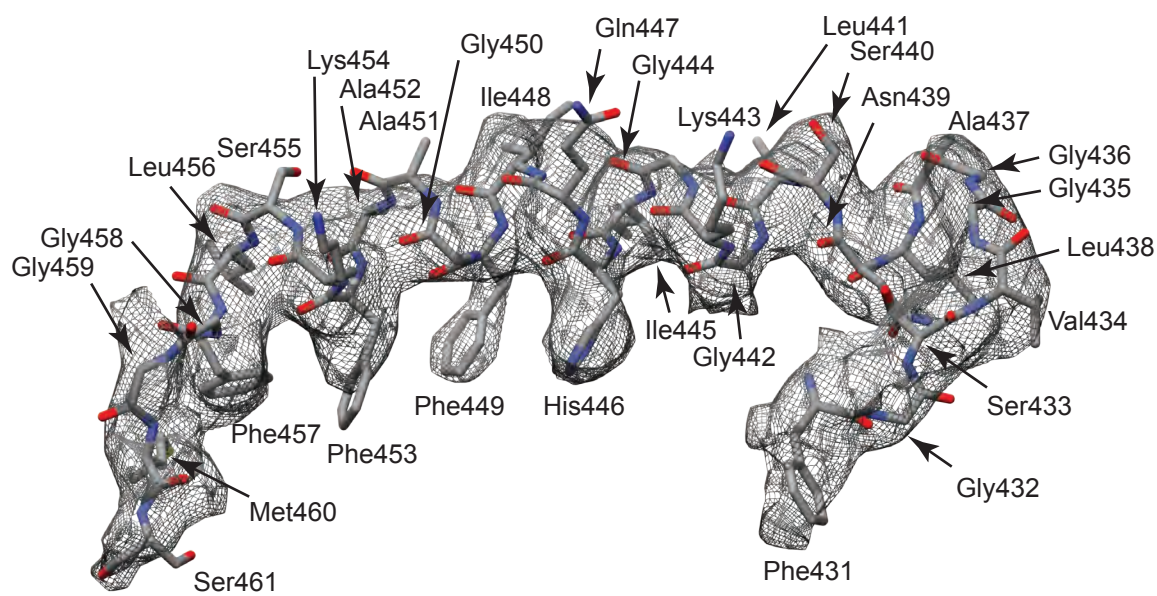

B

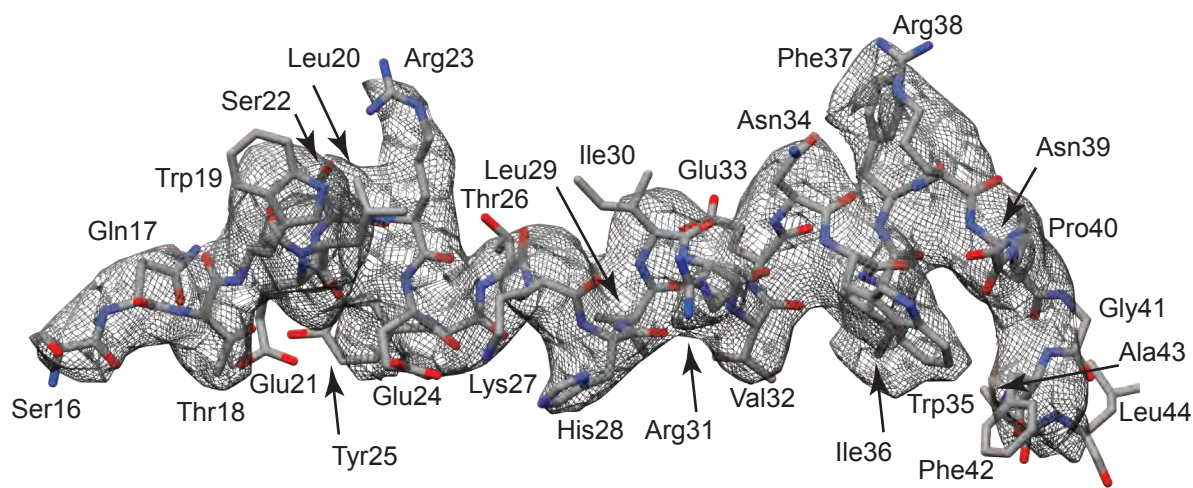

C

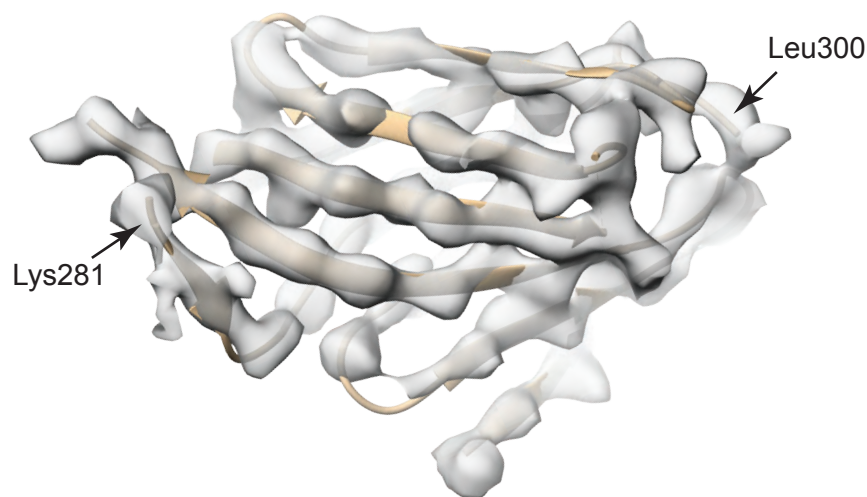

Supplement: Supplementary data 2 [file mmc2.pdf]

# Fig S5

## A

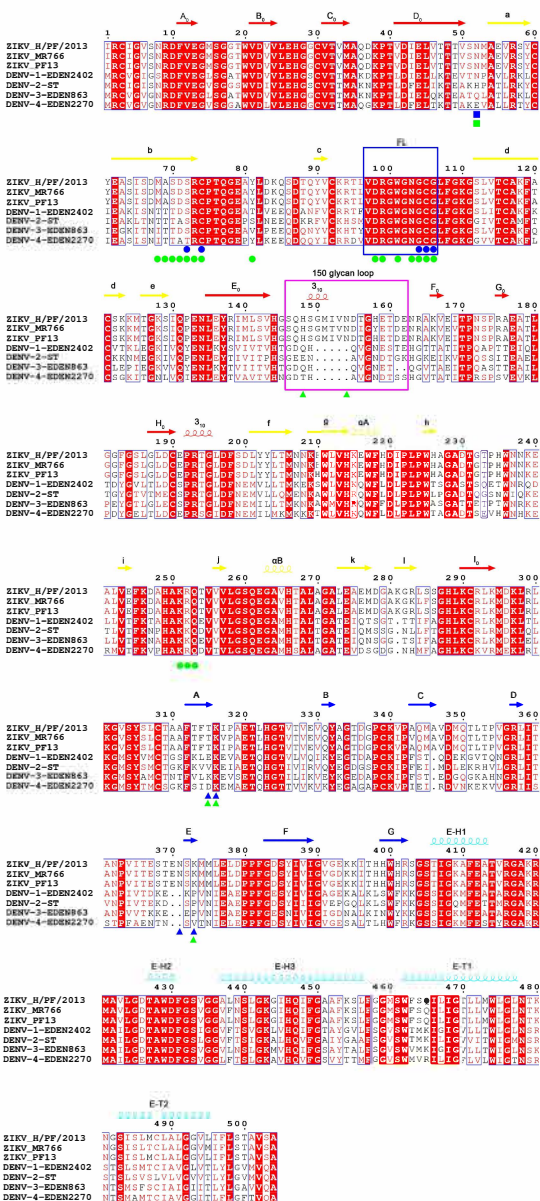

## B

FAB Heavy Chain

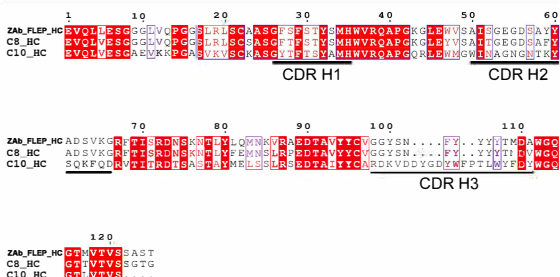

FAB Light Chain

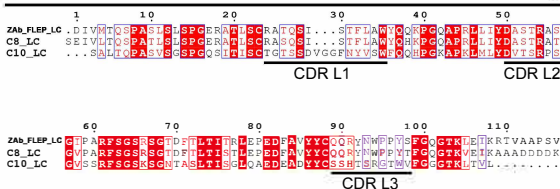

Supplement: Supplementary data 3 [file mmc3.pdf]

# Fig S6

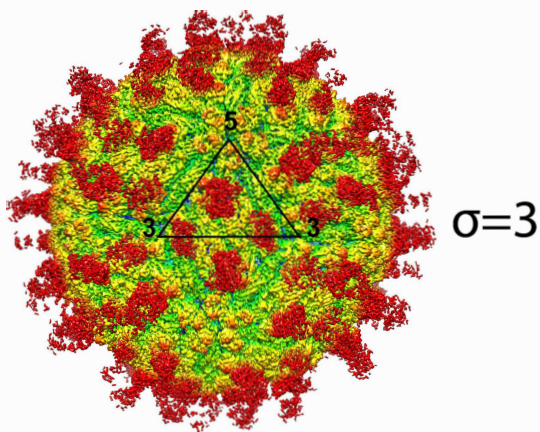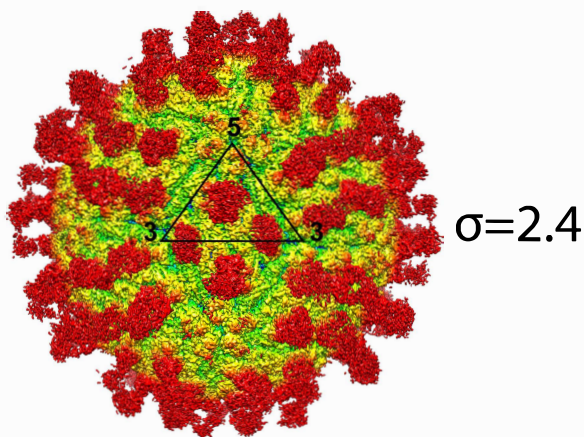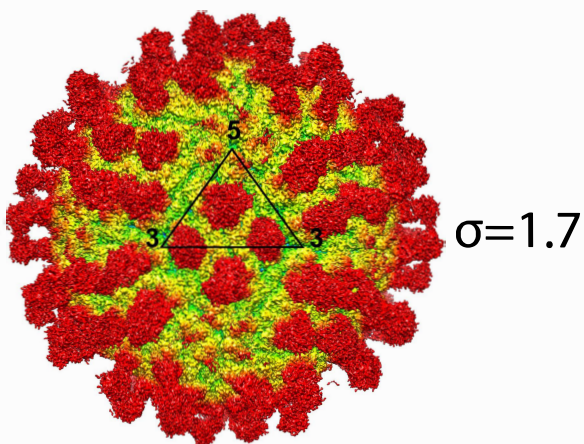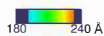

Supplement: Supplementary data 4 [file mmc4.pdf]

Figure S7

## 2-fold ZAb\_FLEP : Zika E protein interface

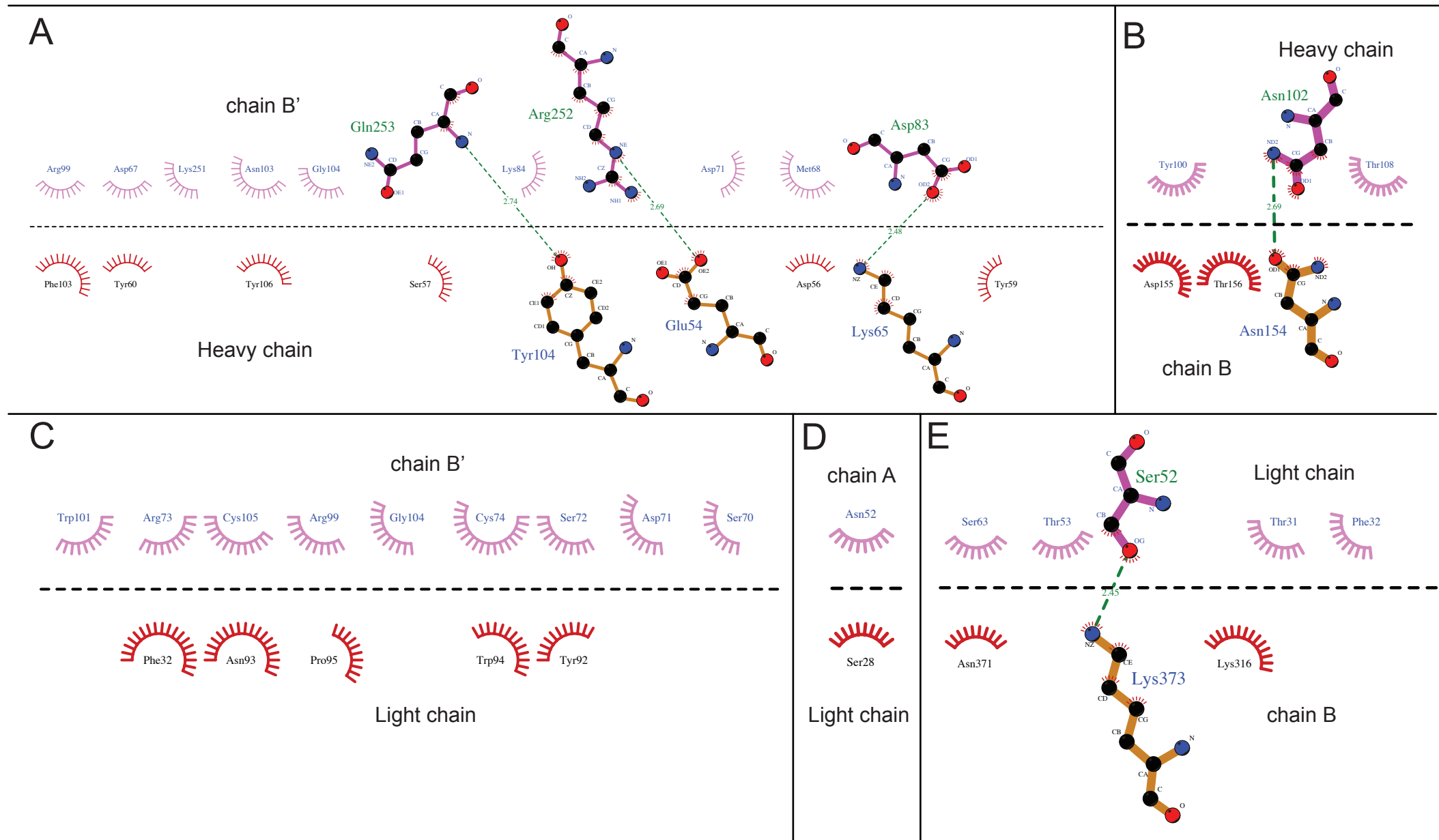

Supplement: Supplementary data 5 [file mmc5.pdf]

### 3-fold ZAb\_FLEP : Zika E protein interface

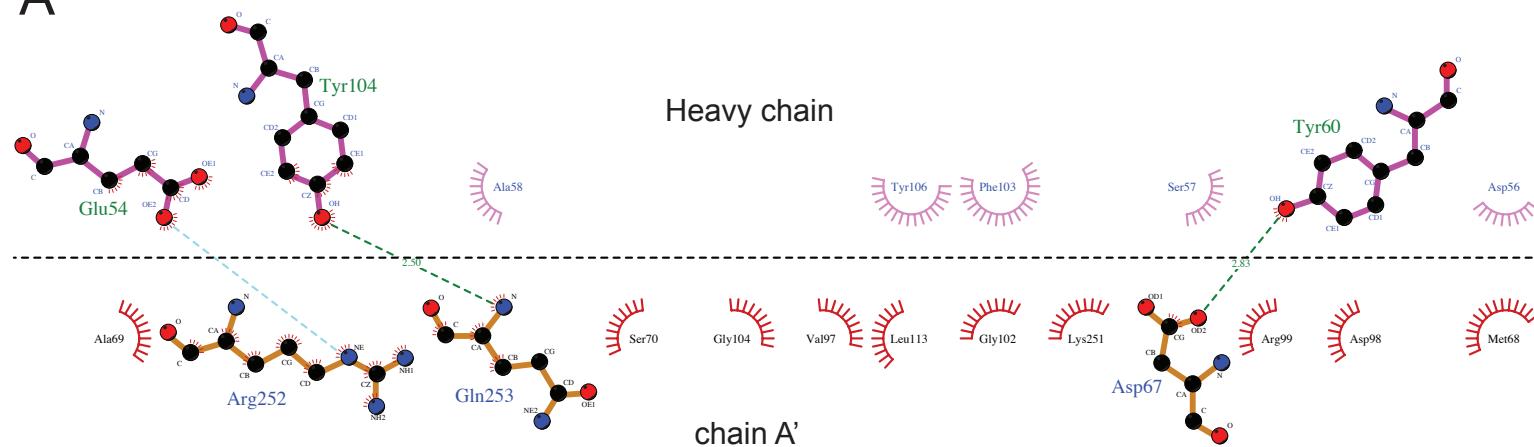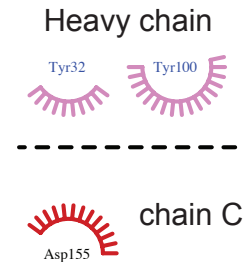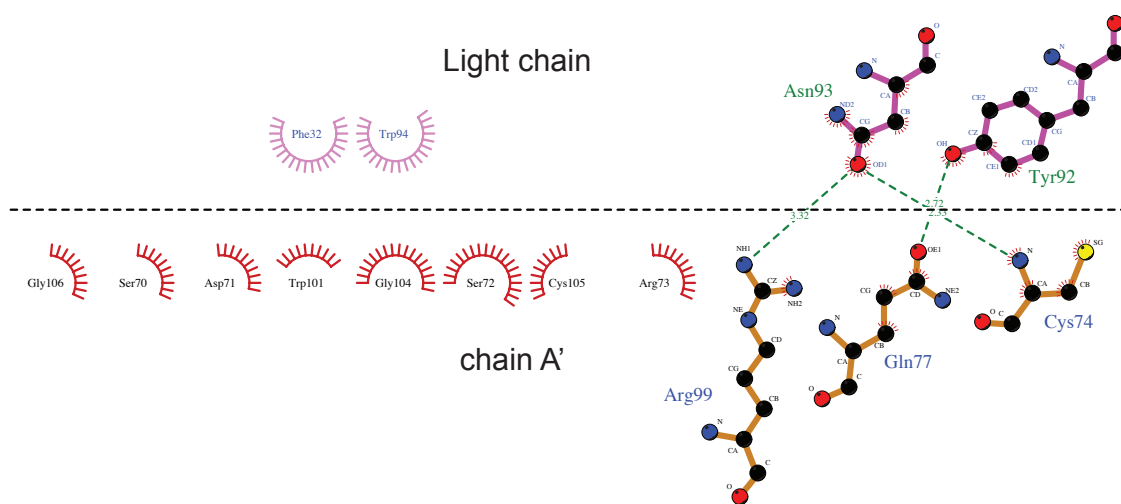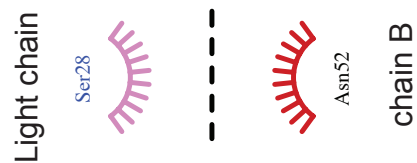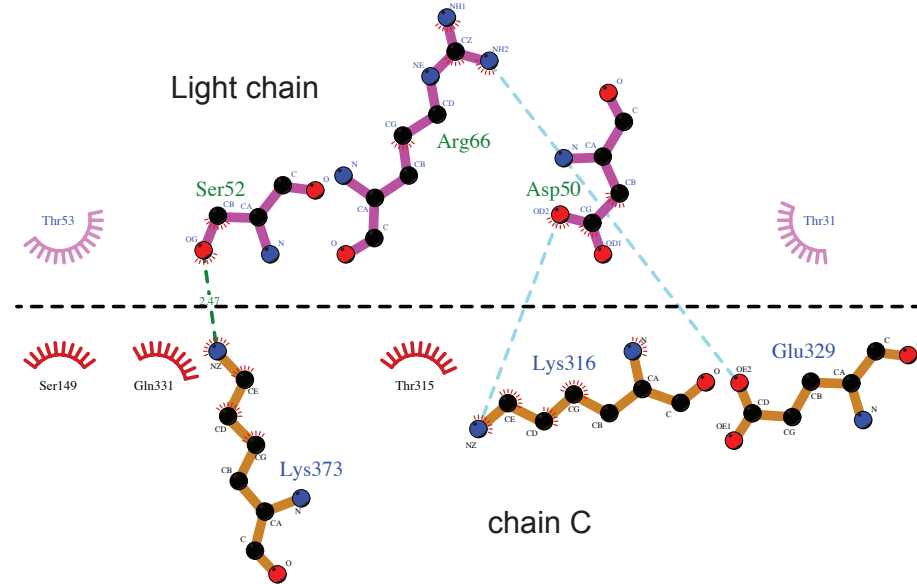

Supplement: Supplementary data 6 [file mmc6.pdf]
