# Supplementary material for: A complex between the Zika virion and the Fab of a broadly cross-reactive neutralizing monoclonal antibody revealed by cryo-EM and single particle analysis at 4.1 Å resolution
Source: J Struct Biol X. 2020 Jun 17;4:100028. doi: 10.1016/j.yjsbx.2020.100028 (PMC7337043; doi:10.1016/j.yjsbx.2020.100028)
Supplement: Supplementary data 7 [file mmc7.docx]

**Table S1. Cryo-EM data and model refinement statistics**

| **Data collection and processing** |  |
| --- | --- |
| No. of micrographs | 7,222 |
| Pixel size (Å per pixel) | 1.045 |
| Total dose (e^-^/Å^2^) | 48 |
| Defocus (µm) | 0.7-3.5 |
| No. of particles used in reconstruction | 4,610 |
| Resolution (Å), FSC=0.143 criterion | 4.1 |
| Map sharpening B-factor (Å^2^) | -99 |
|  |  |
| **Model refinement** |  |
| Correlation coefficient between model and map | 0.78 |
| No. of amino acid residues | 2527 |
| Average B-factor (Å^2^) | 109.9 |
| EMRinger score | 1.61 |
| All atom clashscore | 4.77 |
| Molprobity score | 2.29 |
| RMSD with respect to idealized values |  |
| Bond lengths (Å) | 0.004 |
| Bond angles (°) | 0.713 |
| Ramachandran plot |  |
| Favored (%) | 93.22 |
| Allowed (%) | 6.78 |
| Outliers (%) | 0 |
| Rotamer outliers (%) | 6.38 |
| CaBLAM outliers (%) | 5.50 |
